# Can You Identify This Lesion Seen in a Patient With Melanoma?

**DOI:** 10.6004/jadpro.2012.3.4.10

**Published:** 2012-07-01

**Authors:** Peg Esper

**Affiliations:** From University of Michigan Comprehensive Cancer Center, Ann Arbor, Michigan

## History

**Figure 1:**
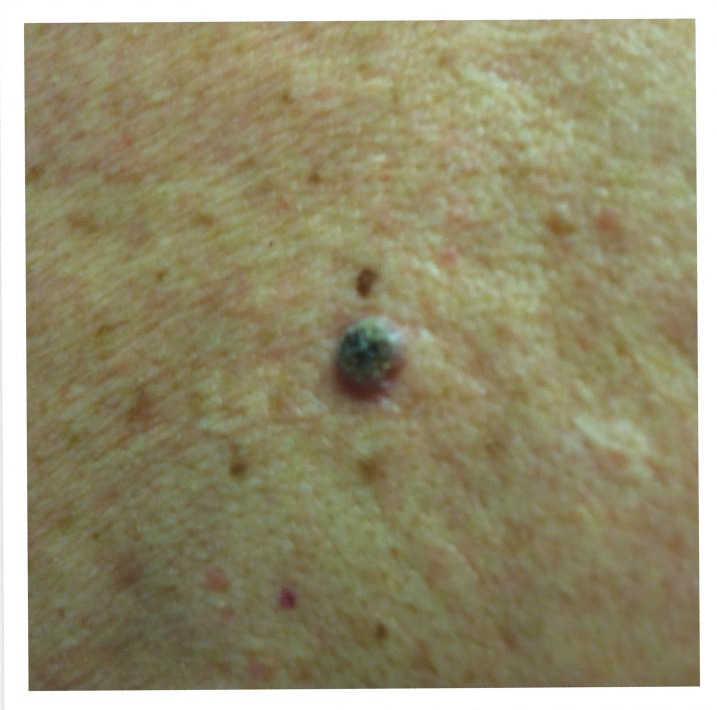


As Mr. V., a 52-year-old male, was getting his hair cut, his barber noticed a dark bump in an area on the side of his scalp, where the hair was thinning. At Mr. V.’s next haircut, his barber indicated that the area was getting larger and that it looked like it had bled. Mr. V. made an appointment with his primary care doctor, who then sent Mr. V. to a dermatologist. The dermatologist biopsied the lesion, which was found to be a 2.8-mm Breslow depth nodular melanoma. Additional characteristics included positivity for angiolymphatic invasion, a positive deep margin, and 12 mitoses/mm^2^.

Mr. V. underwent wide local excision of the lesion with sentinel lymph node mapping. One of two sentinel lymph nodes in the right posterior cervical chain was positive for melanoma, involving approximately 5% of the node. A formal right radical neck dissection was done, and an additional 2 of 22 lymph nodes were found to be positive for melanoma at that time. Adjuvant interferon therapy was initiated postoperatively.

## Chief Complaint

At the time of a regularly scheduled visit 6 months into therapy, Mr. V. presented with increasing anorexia and new abdominal discomfort. He had lost 8 pounds over the previous 6 weeks. On physical exam, a new subcutaneous nodule in the right abdomen and another in the right thigh were noted. A fine-needle aspiration of the abdominal lesion was positive for recurrent melanoma. Subsequent CT scans of the chest, abdomen, and pelvis, as well as an MRI of the brain, included evidence of tumor recurrence in the liver as well as several suspicious pulmonary nodules. Enlarged contralateral lymph nodes were also noted in the neck at that time. A biopsy of the liver was also done, which confirmed stage IV melanoma. The patient’s tumor was sent for molecular testing and a *BRAF* V600E mutation was identified.

Mr. V. was provided with several treatment options, but based on his current symptoms and BRAF tumor status, he elected to start therapy with vemurafenib (Zelboraf) 960 mg orally twice daily. At his 2-week evaluation after beginning treatment, he identified some mild erythema of the skin, which was nonpruritic. He also had some mild fatigue. No dose adjustments were made at that time.

At his next follow-up appointment 2 weeks later, Mr. V. presented with several raised skin lesions on his legs, arms, and back. He noted a sudden onset of these lesions, which were not pruritic and did not bleed but had a crusted appearance. One of these lesions is pictured in Figure 1. Mr. V. was referred back to dermatology for excision of this lesion.

**How would you diagnose the lesion seen in Figure 1?**

- A: *Keratoacanthoma*
- B: *Mycosis fungoides–like eruption*
- C: *Papulopustular rash*
- D: *New primary melanoma*

**Scroll down for correct answer.**

## Correct Answer **A**

**Keratoacanthoma.** An unusual but frequently seen side effect in many patients being treated with BRAF inhibitor therapy is the development of new squamous cell carcinomas of the keratoacanthoma type. In the clinical trials that led to the approval of vemurafenib, this finding was seen in as many as 30% or more of patients undergoing therapy (Flaherty et al., 2010; Lacouture, O’Reilly, Rosen, & Solit, 2012). While this type of malignancy is typically not invasive, it is clearly a frightening side effect for patients who are being treated for a poor-prognosis malignancy such as advanced melanoma.

Since these lesions were first identified in patients undergoing BRAF inhibitor therapy, further research has been ongoing to better identify the etiology associated with this adverse event. It has been shown that many of these lesions are also found to have *HRAS* mutations and often occur in sun-damaged areas of skin. As a result, it is thought that the BRAF inhibitor may not induce these lesions de novo as much as it may serve to exacerbate progression in precancerous lesions within the skin of these patients (Su et al., 2012). Further research suggests that this side effect may be avoided by concomitant use of MAPK/ERK kinase inhibitor therapy; this hypothesis is under clinical investigation (Infante et al., 2011).

While they are very disconcerting to patients, these lesions are typically managed by surgical resection (Robert, Arnault, & Mateus, 2011). Patients should be instructed to bring these lesions to the attention of their clinician once they are identified so that prompt referral can be made to a dermatologist for ongoing management. As these lesions can become numerous, ongoing dermatologic management is critical.

## Explanation of Incorrect Answers

**Mycosis fungoides** is the most commonly seen cutaneous T-cell lymphoma, occurring at a 50% incidence. These lesions may occur singularly but are often seen as multiple erythematous plaque–appearing lesions that subsequently progress into papular nodules. They may be treated with radiation if they cannot be completely excised (Ally et al., 2012).

**Papulopustular rash**, a common dermatologic toxicity seen with many targeted agents, is typically found in areas where the greatest density of sebaceous glands exist. It usually manifests in the form of pruritic papules and pustules (Balagula et al., 2011).

While patients with a primary melanoma are at risk to develop second primary melanomas at a higher rate than individuals who have never had a melanoma skin lesion, this would not be the first suspect in the diagnostic differential for this patient with stage IV disease being treated with a BRAF inhibitor. The crusty top on this lesion with a papular appearance would not be typical for a melanoma lesion.

## Follow-Up

Mr. V. was referred to his dermatologist, who resected several of the suspicious lesions that were pathologically confirmed to be keratoacanthomas. He has subsequently been seeing dermatology every 3 to 4 weeks for skin evaluation and the removal of additional skin lesions. The frequency and number of new skin lesions has been decreasing over the 4 months that Mr. V. has remained on therapy.
